# A comparison of two systems for group housing of gestating sows - effects on productivity, removal, and treatments

**DOI:** 10.1186/s40813-024-00410-9

**Published:** 2025-01-06

**Authors:** Kaisa Ryytty Sylvén, Torun Wallgren, Pontus Almerheim, Lena Eliasson Selling, Magdalena Jacobson, Per Wallgren

**Affiliations:** 1 Farm & Animal Health, Uppsala, Kungsängens gård 731 43 Sweden; 2 Department of Animal Environment and Health, Swedish University of Agricultural Sciences (SLU), Box 7068, Uppsala, 750 07 Sweden; 3 Department of Clinical Sciences, Swedish University of Agricultural Sciences (SLU), Box 7054, Uppsala, 750 07 Sweden; 4 Department of Animal Health and Antimicrobial Strategies, National Veterinary Institute (SVA), Uppsala, 751 89 Sweden

**Keywords:** Pig, Gestation group housing, Liveborn, Floor space, Loose housed sows

## Abstract

**Background:**

Group housing of sows has been extensively studied since the EU banned gestation crating. Well-managed group-housing promotes sow welfare, but the impact varies based on factors such as feeding, group characteristics, and environmental features. Adequate floor space, particularly directly post-mixing, is crucial for social interactions, natural behaviours, and to reduce injuries caused by aggression. The aim of this study was to compare two group-housing systems for gestating sows with respect to productivity, treatment frequency, and removal of sows. Both systems were static but differed in space allowance, quantity of enrichment material and feeding management. System I comprised of large sized pens with deep litter straw bedding, housing in total 40 sows, and System II of smaller sized pens with permanent access to straw, housing 8 to 10 sows.

**Results:**

The mean parity number was 3.1 ± 1.3 in both groups. Sows housed in System I with large groups (*n* = 40) in large pens with deep litter straw gave birth to 16.8 ± 0.33 (Least Squares Means, LSM) piglets, compared to 15.4 ± 0.31 (LSM) for sows in System II kept in smaller groups (*n* = 8–10) in smaller pens (*p* = 0.0005). Medical treatments of sows were more frequent (*p* < 0.001) in System II. The incidence of replacement of sows was comparable in both systems, and there was a high occurrence of sows becoming pregnant during the subsequent insemination in both groups.

**Conclusions:**

This study indicated that sows kept in larger groups provided with a larger floor space (a total area of 156 m^2^, corresponding to 3.9 m^2^ per sow) and housed on deep straw had a higher number of liveborn and weaned piglets and lower incidence of antibiotic treatments than sows with less floor space (a total area of 24.5 m^2^, corresponding to 2.5–3.1 m^2^ per sow) and less bedding/manipulable material.

## Background

Group housing of sows is currently an extensively studied topic, since the EU banned the crating of sows throughout the gestation period [1]. Studies have explored how housing impacts welfare and productivity, with a consensus emerging that group housing promotes sow welfare [2, 3]. For example, sows in well managed group-housing systems exhibit increased relaxing behaviour and decreased stereotypies [4].

However, the impact of different group-housing systems on sow health continues to be a complex area of study [5, 6]. Stressors may arise from factors such as feeding system or features related to the feed (e.g., fibre content), group characteristics, floor characteristics and environmental enrichments [2, 3, 6–8]. Stress during particularly sensitive phases, such as around fetal implantation or the peri-parturient period, can significantly impact a sow’s health and productivity. The duration of the stress is also crucial; while sows can generally handle stress lasting less than a day, stress persisting for more than two days (chronic stress) can have adverse effects [9].

Floor space is essential for social interactions, hierarchy establishment, and to enable natural behaviour [10]. Increased floor space has been identified to alleviate stress caused by aggression during regroupings/mixing of sows [3, 5, 11, 12]. Notably, increased floor space appears to be most critical immediately post-mixing [9, 12]. Consequently, management strategies with increased floor space at mixing therefore emerges as a viable option [11, 12]. Adequate floor space also supports exercise, maintain muscle tone, and improve bone composition and strength [10, 13, 14]. However, it is difficult to draw clear conclusions on the minimum floor space requirements as the quality of the pen (e.g., space for subordinate sows to avoid conflict, flooring etc.), the total space shared by the sows, and management strategies are as important. Still, studies have suggested that 1.4 m^2^ per sow is inadequate [3, 15] whereas > 3 m^2^ sow reduces aggressive interactions and has positive effects on litter size [16].

The social hierarchy typically stabilizes within 24 h post-mixing, after which aggressive behaviour decreases [9]. In systems where sows are transferred to mating units and group-housed immediately after weaning, the social hierarchy will therefore generally establish before mating. Establishing a well-defined social hierarchy prior to admission to the gestation unit reduces post-mating stress [6, 9, 17, 18]. Effectively managing groups of sows after weaning can alleviate adverse effects on fertilization and implantation caused by elevated cortisol levels resulting from stress [9]. Consequently, persistent stress during pregnancy in sows could have detrimental effects on foetal development and increase the likelihood of abortions [18].

Group characteristics during gestation may influence sow health. While dynamic groups allow sow removals and introductions [19], they are more prone to chronic stress than static sow groups [20]. Establishing static groups at weaning or the beginning of gestation, without replacements if a sow is removed, can prevent stress. In both systems space allowance is of importance as it facilitates for subordinate sows to evade conflicts. Consequently, large static groups may provoke fewer injuries than smaller dynamic groups, due to a larger total floor space and a stable social hierarchy [21].

The EU Directive 2008/120/EC outlines minimum standards for pregnant sows [1] (Table 1). According to the EU legislation, sows must be group-housed from four weeks after farrowing until one week before expected farrowing. In Sweden, the use of sow crates has been banned since 1988 [22]. Consequently, various systems and management strategies for housing pregnant sows have been developed. The aim of this study was to compare productivity, treatment frequency, and removal of sows in two commonly used Swedish group-housing systems for pregnant sows applied on one farm, thus employing identical feed and management strategies.

**Table 1:** A comparison of items in the EU legislation concerning the welfare of sows and gilts and conditions for sows and gilts in the studied herd

Parameter	Mating unit (deep straw bed)	Gestation System I (deep straw bed)	Gestation System II (pen)	Reference EU- legislation
Minimum unobstructed floor space per gilt/ sow	3.9 m^2^	3.9 m^2^	3.1 m^2^; 8 sows 2.5 m^2^; 10 sows	Gilts after service > 1.64 m^2^ Gilts and sows in groups 2.25 m^2^ < 6 animals: + 10% > 40 animals: − 10%
Solid floor for gestating gilts and sows	3.9 m^2,^	3.9 m^2^	2.4 m^2^; 8 sows 1.9 m^2^; 10 sows	> 0.95 m^2^ per gilt > 1.3 m^2^ per sow
Drainage opening	-	-	-	Max 15%
Slats for gestating gilts and sows	-	-	Gap width: 20 mm Slat width: 80 mm	Gap width: 20 mm Slat width: 80 mm
Manipulable material	Straw bedding	Straw bedding	Straw: 0.75–0.94 kg per day and sow	Permanent access for sows and gilts
Feed	Free access stalls, physical separation during feeding, liquid feeding system	Free access stalls, physical separation during feeding, liquid feeding system	Drop feeding, feeding area per sow: 0.22–0.27 m^2,^ no physical separation during feeding, liquid feeding system	Sufficient of bulky high-fibre food as well as high-energy food for each individual
Feeding	Twice a day	Twice a day	Twice a day	At least once per day
Drinking water	Permanent access. Two cups/40 sows	Permanent access. Two cups/40 sows	Permanent access.Nipple drinker	Permanent access to fresh water
Water flow	4 L per minute	4 L per minute	4 L per minute	-
Diseased/ injured pigs in group housing	Sick bay available	Sick bay available	Sick bay available	May be housed individually in sick bay, should be able to turn around
Continuous noise levels	< 80dB	< 70dB	< 75dB	< 85 dB

## Methods

### Study design

The study was designed as an observational descriptive/explanatory retrospective cohort study in sows that met the inclusion criteria of being housed their entire gestation in large-sized pens with deep litter straw or in gestation pens. The study was conducted retrospectively after observing differences in sow health and productivity that appeared to be linked to their housing conditions during gestation. This prompted a closer examination of production data from sows housed in different conditions.

### Studied sows were only included once in the study

The study was conducted in the central unit of a conventional multisite production herd with 1,800 Landrace x Yorkshire sows (Topigs Norsvin). From February to September 2022, data on farrowing performance, removal, treatment of sows and pregnancy rates were collected and analysed. Sows farrowed either at the central unit or at farrowing sites located elsewhere. To ensure uniform conditions for all factors except housing conditions during gestation, only sows that farrowed at the central unit were included in the study.

**Fig. 1:**
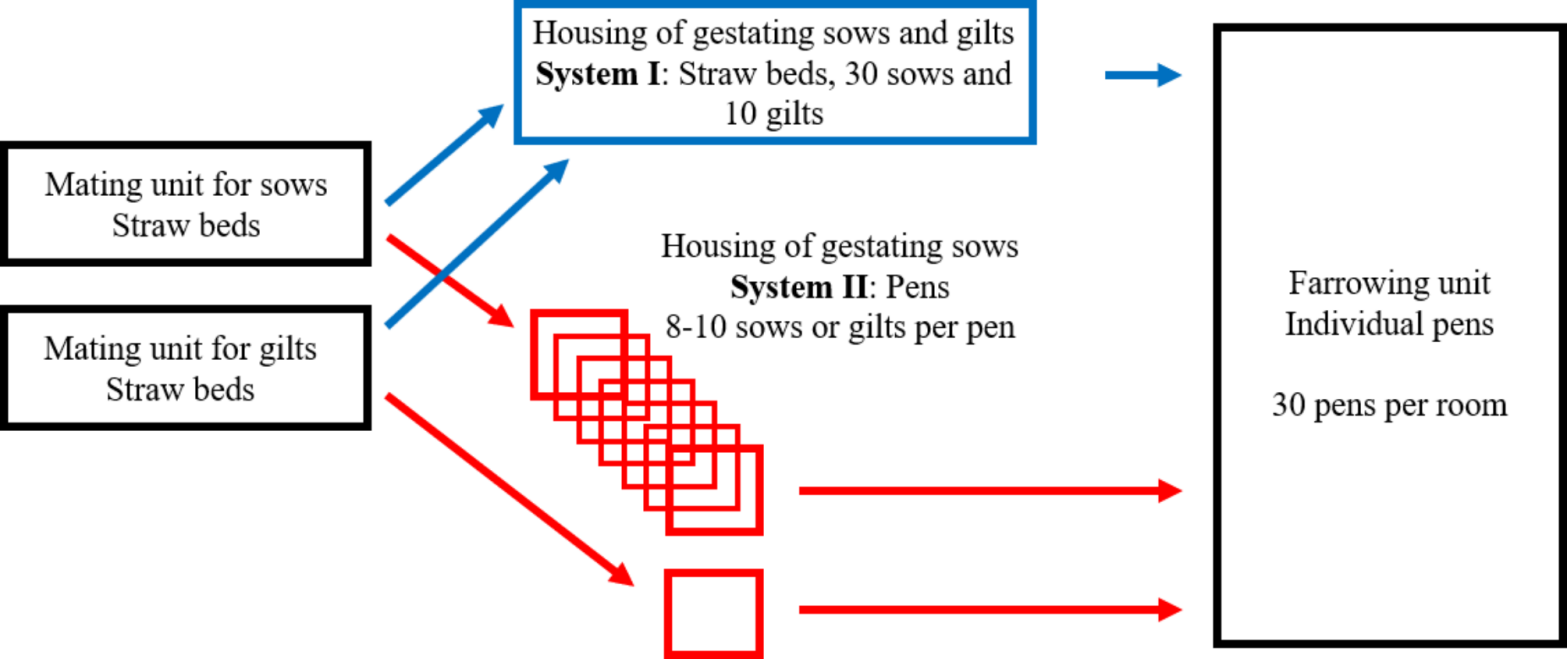
Animal flow of studied sows

Sows entered the mating unit at weaning (mean 34 days post farrowing) and were allocated to the facilities for gestating sows seven days later (Fig. 1). Throughout the gestation period, sows and gilts were either housed in groups of 40 individuals on deep litter straw (System I, *n* = 120, mean parity number 3.0 ± 1.3) or kept in groups of eight to ten sows per pen (System II, *n* = 137, mean parity number 3.0 ± 1.3). In both gestation systems the sows were kept in static groups.

Figure 1. During mating, dry sows and gilts were kept in identical straw beds with 40 animals per unit (black boxes). In System I, mated sows were transferred to pens with deep straw bedding where 30 sows were mixed with 10 recently mated gilts (blue boxes). In System II, mated sows were transferred to pens with 8–10 sows/pen. Gestating gilts were reared in separate but identical pens (red boxes). Sows and gilts were transferred to individual farrowing pens five days prior to farrowing.

All sows received a liquid feed in a system where the dry components of the feed were mixed with the wet part immediately before feeding, leaving no residues in the feeding pipes between feedings. The composition of feed was identical for all sows of the same production stage. The animals were cared for by the same staff throughout the study period.

### Housing of dry sows – from weaning until one week after mating

**Fig. 2:**
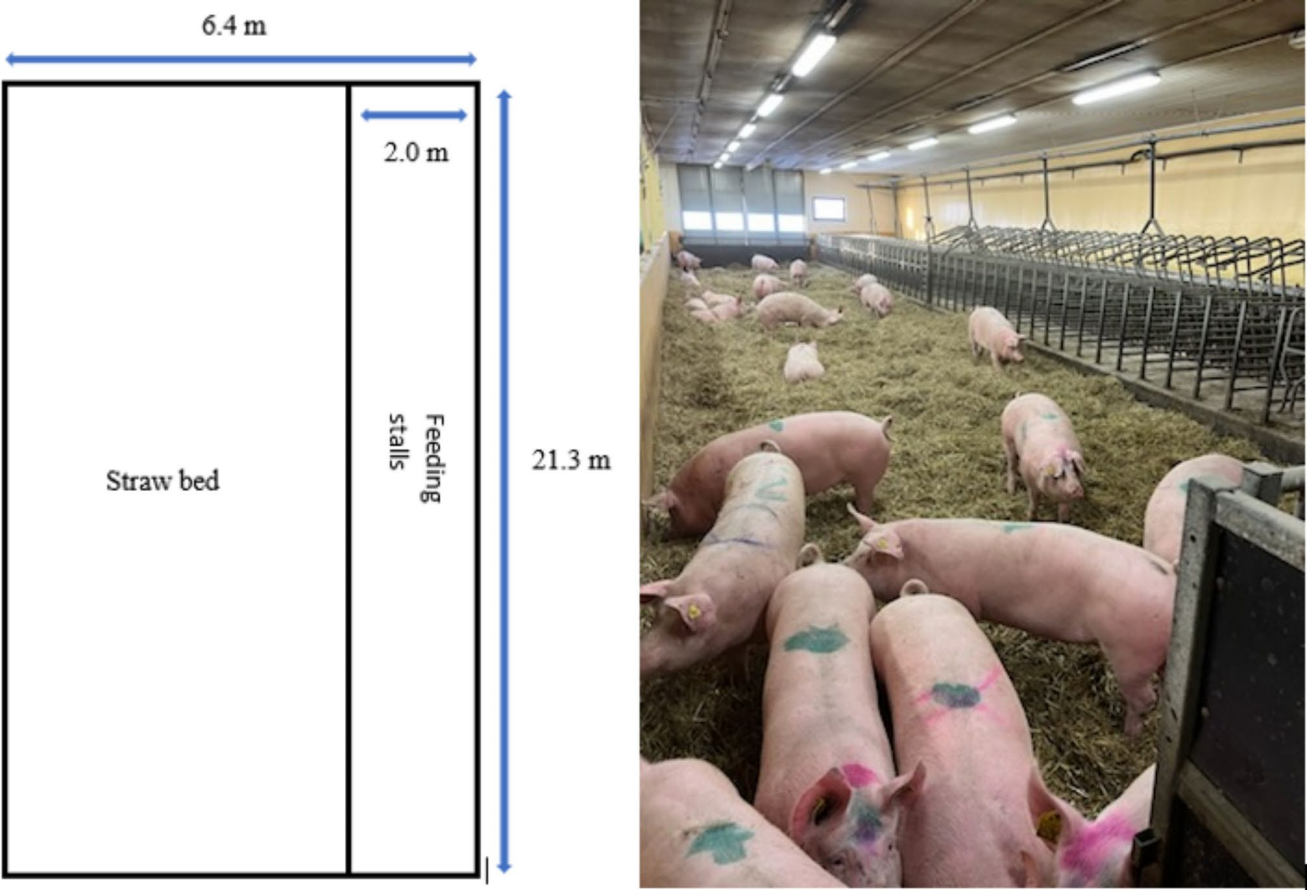
Schematic drawing and photograph of deep straw bed units, for dry sows and System I

At weaning, sows were sorted in groups of 40 on deep straw beds measuring 6.4 by 21.3 m, providing 3.1 m^2^ per sow (Figs. 1 and 2). In addition to this area, the sows had unrestrained access to 40 feeding stalls sized 0.8 m^2^ per sow. Thus, the total area was 156 m^2^, corresponding to 3.9 m^2^ per sow. During the oestrus period (3 to 6 days post-weaning), sows were inseminated with Hampshire semen (Nordic Genetics). Following seven days in the mating unit, the entire group of sows was relocated either to a gestation unit with deep straw bedding (System I) or to a unit with gestation pens (System II).

### Housing of gestating sows, System I – from one week after mating

System I comprised deep straw bed units in un- insulated buildings with free access stalls accommodating approximately 30 sows and 10 gilts (Figs. 1 and 2). The recently mated sows were integrated into these facilities along with recently mated gilts. Following the introduction of gilts to the group, the group was kept static.

The deep straw bed, situated on concrete floor, offered an area of 3.1 m^2^ per sow with feeding stalls providing an additional 0.8 m^2^ per sow excluding feeding troughs, corresponding to a total area of 156 m^2^ and 3.9 m^2^ per sow. The straw bed was initially at least 10 cm thick and gradually reached up to 40 cm by the end of the gestation period as new straw was replenished once or twice weekly, depending on climate conditions and straw bed hygiene. The entire straw bed was removed, and the units were cleaned and disinfected between batches.

Sows were fed a liquid diet, each feeding tube served four troughs sized 0.45 by 0.30 m. Sows were fed twice daily with a two-hour interval, and during this time, they were confined in the feeding stalls with the aim to prevent feed stealing by sows of high social rank. Additional individual feeding was tailored based on the body condition of each sow, involving extra dry feed provided manually in the trough when the sows were confined. Body condition scoring was made through visual assessment of each sow. Water was supplied through water cups, with two cups allocated per straw bed unit. Ventilation was regulated through natural means, ensuring compliance with legislation [1] for air quality.

### Housing of gestating sows, System II – from one week after mating

**Fig. 3:**
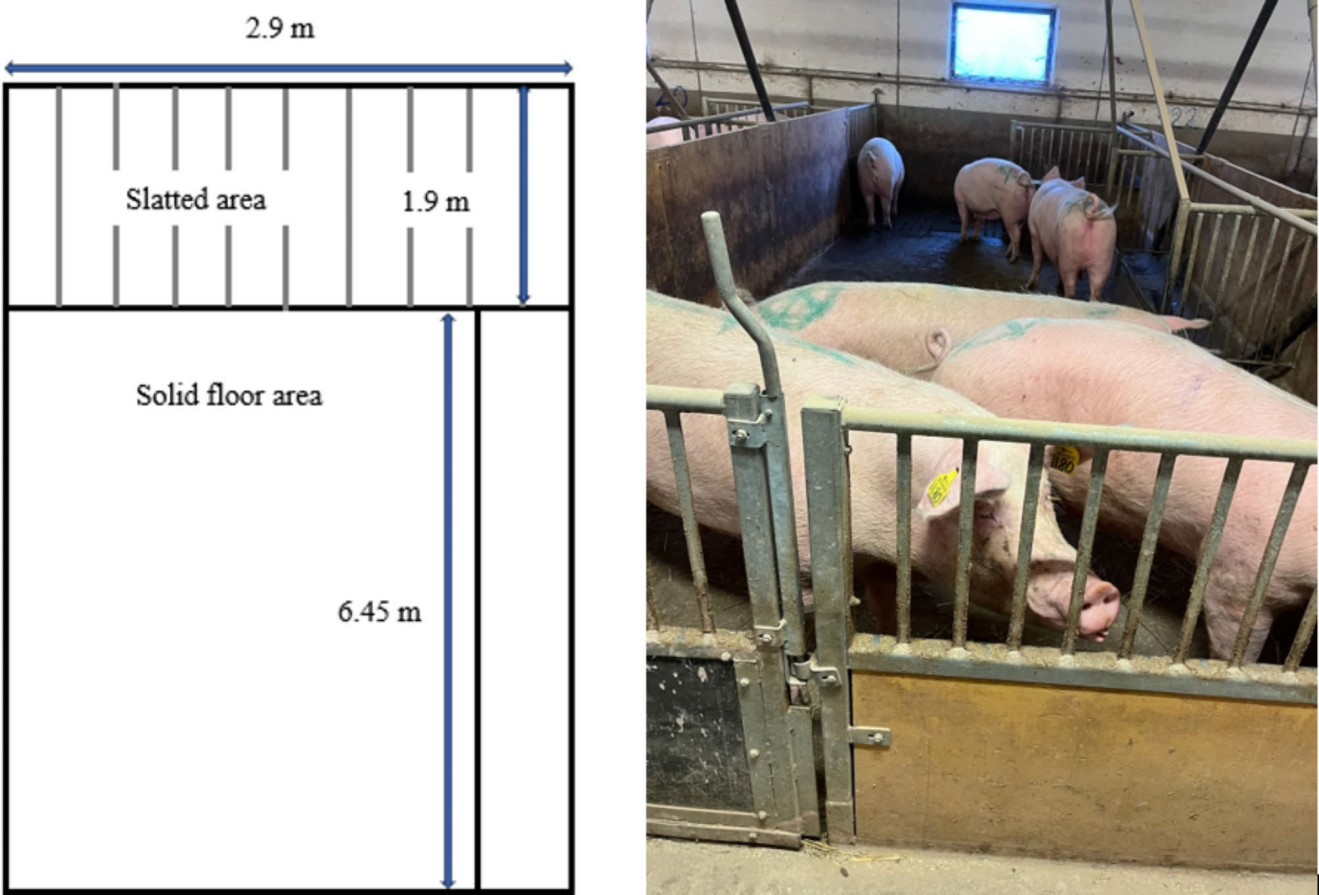
Schematic drawing and photograph of housing of gestating sows in System II

System II featured pens accommodating eight to ten sows or gilts (Figs. 1 and 3). Upon transition from the mating unit to the gestation pens, eight to ten sows were sorted based on size and body condition, i.e., small sows of inferior condition were grouped together. The system maintained static groups throughout gestation. Gilts were reared alongside with the sows in identical pens and joined the sow group during the second gestation. Each pen was cleaned and disinfected between each batch.

The pens comprised a solid concrete area measuring 6.5 by 2.9 m (1.9 to 2.4 m^2^ per sow) and a dunging area with slatted floor sized 1.9 by 2.9 m (0.65–0.7 m^2^ per sow), the slats were 8 cm wide and were separated by 2- cm slots. Thus, the total area was 24.5 m^2^, corresponding to 2.5–3.1 m^2^ per sow/ gilt. Liquid feed was provided by drop feeding [23]. The trough, that measured 6.45 m in length and 0.34 m in width, offered a feeding area of 0.22–0.27 m^2^ per sow. No individual extra feeding was conducted in this system. Sows had free access to a nipple drinker. Straw, complying with the EU and Swedish legislation, was provided daily at a rate of 7.5 kg per pen (0.75–0.94 kg per sow), ensuring permanent access to manipulable material [1].

The concrete floor had undergone renovation 10 years ago and the slurry system employed was a liquid system managed once daily. Ventilation was maintained through negative pressure in a mechanical ventilation system, ensuring compliance with air quality regulations [1].

### Housing of farrowing sows

The farrowing facilities consisted of six identical units. Each farrowing unit was equipped with 30 pens, each sized at 6.8 m^2^ and designed for free farrowing. “All in, all out” management was practiced and after each batch, the units were emptied, thoroughly cleaned, and disinfected.

Sows entered the farrowing pens five days before anticipated farrowing. To facilitate nest building, 3–4 kg of straw per sow were provided 48 h before the expected farrowing. Within batch, the sows farrowed over approximately one week, and the nursing period averaged 33.9 ± 4.8 days.

### Parameters registered

The study included 120 sows and gilts from (seven groups/ pens) in gestation System I and 138 sows and gilts from (16 groups/ pens) in gestation System II. The study exclusively considered sows that gave birth at the central unit, ensuring consistent management practices for all sows during lactation.

Data collected included farrowing performance on sow level (total born, liveborn, stillborn and weaned piglets), as well as pregnancy rates at the subsequent insemination. Cross-fostering was practiced between sows in the same farrowing room but no registration of moved piglets were made in the management software program.

The study incorporated data on sow mortality, encompassing instances of sows found dead, euthanized, and removed sows, along with the reasons behind euthanasia or the decision for removal.

Additionally, treatments of sows with antibiotics and non-steroidal anti-inflammatory drugs (NSAIDs) were documented during both the gestation and nursing periods. Data were recorded using a pig management software system, specifically AgroVision’s PigVision [24].

### Statistical analysis

All data was collected on sow individual level per farrowing and subsequently transferred to Microsoft Excel for descriptive analysis. Some descriptive analysis and statistical analyses were further analysed using SAS 9.4 (SAS Inst. Inc., Cary, NC). Descriptive statistics were calculated on housing level based on individual sow data. The effect of housing on the different production variables was investigated through analysis of variance as described below.

Removal of sows, antibiotic treatment, and total number of treatments (Antibiotic or NSAID) was investigated using the Chi-square test and through calculating relative risk comparing the different housing systems.

To analyse the effect of housing on the number of piglets born alive, weaned piglets, and pregnancy rate (normally distributed variables) a general linear model (proc glm) was used to construct a statistical model accounting for housing, parity, season and the interaction between housing and parity using backward elimination. To analyse the effect of housing on the number of stillborn piglets (non-normally distributed), a Poisson regression model (proc genmod) with log transformation was used to construct a statistical model accounting for housing, parity, season and the interaction between housing and parity using backward elimination. Three seasons were created: Jan-Feb, March- April, May- June. Season was then excluded from the models due to not being significant. Comparisons were made through Least Squares Means (LSM).

Information on cross-fostering were not registered and therefore, piglet mortality was not possible to analyse at litter level. Further, not all sows in each farrowing group conformed to the inclusion criteria and were hence not included in the study, and therefore analysis at group or batch level was also impossible.

To investigate the difference in performance between gilts and sows within housing system, production means (number of totally born, liveborn, stillborn and weaned piglets) were compared by a t-test.

## Results

### Productivity of sows

As seen in Table 2, the parity number ranged from one to nine (mean 3.0 ± 1.3 in both groups).

**Table 2:** Distribution of parity number for gestations in System I and System II. The table also shows the overall Least Squares Mean number of piglets born alive per parity number

	System I (Straw bed)	System II (pen)	Sows and gilts (both systems combined)		
	(*n* = 120)	(*n* = 138)	(*n* = 258)	Liveborn (LSM)	
Parity 1	15	1	16	13.1	A
Parity 2	30	63	93	16.2	B
Parity 3	30	36	66	16.7	B
Parity 4	29	23	52	16.7	B
Parity 5	13	7	20	15.4	AB
Parity 6	3	7	10	14.3	AB
Parity 7	0	0	0	-	-
Parity 8	0	0	0	-	-
Parity 9	0	1	1	14.8	AB
**Mean ± SD**	**3.0 ± 1.3**	**3.0 ± 1.3**	**3.0 ± 1.3**		

Table 2. Different letters after Least Squares Mean-values indicate statistically significant difference in number of liveborn piglets between parities. The results are based on a general linear model including housing system and parity number. Parity 1 differs from all parities except parity 5, 6 and 9. Since data regarding parity was unbalanced between the housing systems only combined data from both systems were used to calculate LSM for liveborn per parity.

The mean number of piglets born alive in parity 1 differed from parity number 2, 3 and 4 (*p* < 0.001), and the number of piglets born alive increased with parity number up until parity number 5 (Table 2).

As seen in Table 3, sows accommodated in System I on deep straw beds exhibited 1.4 more liveborn piglets per litter in comparison to sows housed in System II with gestation pens. The number of liveborn piglets was significantly affected by housing (System I LSM 16.8, System II LSM 15.4, *p* = 0.0005) and parity (System I LSM 16.1, System II LSM 14.5, *p* = 0.0074). On average, 1.1 piglet was stillborn in both groups (*p* > 1.0). A numerical discrepancy existed in the count of weaned piglets between sows from System I (14.6) and System II (14.0), but this difference lacked statistical significance (T-test, *p* = 0.2003).

**Table 3:** Production parameters in farrowing unit for sows housed in System I and System II

	System I (Straw bed) (*n* = 120) LSM	System II (pen) (*n* = 138) LSM	Housing *p* – value	Parity *p* – value
Totally born*	17.8 ± 3.8	16.6 ± 3.5		
Liveborn	Housing:16.8 ± 0.33 Parity:16.1 ± 0.62	Housing:15.4 ± 0.31 Parity:14.5 ± 0.61	0.0005	0.0074
Stillborn	1.1 ± 0.09	1.1 ± 0.1	NS	0.0001
Weaned	14.6 ± 0.29	14.0 ± 0.27	NS	-

*Arithmetic mean, as no model was created

Table 3. There was a statistically significant difference in the number of liveborn piglets between the gestation systems (Housing) and a statistical significance for parity within the model. For stillborn both housing and parity were included in the model, for weaned only housing was included in the model.

Pregnancy rates during the subsequent mating was 99.4% among sows in System I (*n* = 83 of 84 sows) and 98.6% among sows in System II (*n* = 105 of 108 sows).

Treatments and replacement of sows.

The number of sows that were euthanized, found dead, or removed during the gestation period and up to the next mating is shown in Table 4.

**Table 4:** Euthanized, dead and removed sows including reasons for euthanasia or removal of sows housed in System I and II

	System I (Straw bed)	System II (pen)
	(*n* = 120)	(*n* = 138)
**Number of sows euthanized or found dead**	**5** **(4.2%)**	**5** **(3.6%)**
**Number of removed sows**	**16** **(13.3%)**	**13** **(9.4%)**
**Reasons for euthanasia or removal (excl. sows found dead)**		
Low productivity	8 (6.7%)	5 3.6%)
Injuries, fractures, weakness	3 (2.5%)	5 (3.6%)
Other reasons	4 (3.3%)	6 (4.3%)

*Other reasons for removal: udder health, failed reproductive performance

As shown in Table 5 the number of sows subjected to antibiotic treatment and the overall count of treated sows (including NSAIDs or antibiotics) within the farrowing unit differed between the groups (*p* < 0.0001). Among the sows accommodated in System I, there was a single antibiotic treatment and a total of five treatments (NSAIDs or antibiotics) administered in the gestation unit. Sows housed in System II, underwent four antibiotic treatments during gestation, all treatments in the gestation units were due to arthritis. When housed in the farrowing units there were 29 antibiotic treatments and a total of 52 treatments (NSAIDs or antibiotics) among sows from System II. The relative risk of being treated with antibiotics was nine times higher among sows housed in System II as compared to System I.

**Table 5:** Treatments with antibiotics, NSAIDs and total number of treatments (NSAIDs or antibiotics) for sows housed in gestation system I and II

	System I (Straw bed)	System II (pen)	
	(*n* = 120)	(*n* = 138)	
**During the gestation period**			
Sows treated with antibiotics	1 (0.8%)	4 (2.9%)	
Sows treated with NSAIDS	2 (1.7%)	0	
**Total number of sows treated**	**3** **(2.5%)**	**4** **(2.9%)**	
**During the nursing period**			Significance
Sows treated with antibiotics	1 (0.8%)	29 (21.0%)	*p* < 0.001
Sows treated with NSAIDS	4 (3.3%)	23 (16.7%)	*p* < 0.001
**Total number of sows treated**	5 (4.2%)	52 (37.7%)	*p* < 0.001

## Discussion

The study highlighted significant differences between gestation System I, where 40 pregnant sows were housed on deep litter straw beds, and gestation System II, which accommodated 8–10 pregnant sows in pens. System I exhibited a higher count of both totally born and liveborn piglets, as well as a greater number of weaned piglets, despite potential expectations of a lower number of piglets born alive due to a higher percentage of gilts (12.5% versus 0.7%). Parity number typically influences the number of liveborn piglets, with lower numbers expected in parity one [25, 26]. Sows in System I experienced mixing when gilts were introduced to the group seven days post-weaning. However, sows and gilts in System II also were regrouped when transferred from mating unit to smaller groups in the gestation pens, possibly including the establishment of new social hierarchic orders. Considering the smaller area in System II (pens) with difficulties for subordinate animals to avoid conflicts, a negative impact on the reproductive performance cannot be excluded. If so, this probably occurred in the early phase of gestation, as the incidence of stillborn piglet did not differ between the groups. The study may indicate a positive effect on reproductive performance of group- housing of large static groups in a larger pen with high access to straw.

As almost all sows in both groups became pregnant, no impact of housing on the next gestation was indicated. This was not surprising since the reproductive performance during the early gestation is dependent on the condition of the sow during mating [23], and both groups had been identically managed during farrowing and lactation.

The study found no differences in total sow mortality (euthanized, found dead and removed) between the two systems, but it noted a numerical increase in the incidence of sows removed or euthanized due to mechanical injuries, fractures, and weakness in System II. The smaller area in System II (pens), limited opportunities for subordinate animals to avoid conflicts and limited use of enrichment material could contribute to this, though statistical relationships were challenging to establish due to the limited number of removals and deaths. However, further investigations in a larger study would be intriguing to perform. Further, the groups were not equally distributed in terms of parity number, and there is a lack of knowledge on the overall sow stayability in the different systems, which could have affected the results. In this study, our analysis was limited to the housing conditions of the sows during a single gestation period, making it impossible to account for any potential influence of prior gestation conditions.

System I showed significantly fewer treatments for sows, both in terms of antibiotic treatments alone and the total number of treatments. Although there were minor differences in the number of sows treated with antibiotics during gestation (one treatment in System I and four treatments in System II), all treatments were attributed to arthritis. The risk of lameness in group-housed sows is influenced by various interconnected factors, including those affecting stress [2, 8, 18, 27, 32]. Sows in dynamic groups experience a higher prevalence of lameness and skin lesions [28], and other factors such as sow age also play a role, with a higher incidence of lameness in younger sows [29–31]. Slatted floors may double the odds of lameness compared to solid floors [27], and the incidence of lameness increases with aggression during mixing [16]. The use of deep straw bedding and the larger total area of 3.9 m^2^ per sow in System I, might have contributed to fewer injuries to the locomotor apparatus as compared to the pens in System II, where straw served solely as enrichment and the floor was partly slatted. The flooring and bedding material in System I might have mitigated potential injuries and the opportunity for exercise and avoiding aggression was greater in System I. Exercise, increased bone density, improved piglet survival, and altered lying behaviour among gilts have been linked to good leg health, enhancing animal welfare and productivity [13, 14, 31]. Despite System I including more gilts than System II, the instances of arthritis treatment and removal for fractures, injuries, and weakness were lower in System I which may be reflected in the higher number of total and liveborn piglets from sows in System I.

As mentioned earlier a larger space allowance positively affects sow exercise and muscle tone of the animal [10, 16], preparing them for physically and psychologically stressful events, such as movement to the farrowing unit and the farrowing process [32]. Staff observations indicated that sows from System I were easier to move, more alert at feeding times in the farrowing room, and generally more active. This alertness might also have influenced the nest-building behaviour performed by the sows. Nest-building behaviour, along with feed and water intake, has been recognized for its positive impact on farrowing outcomes [33, 34] which may be reflected in the higher number of weaned piglets from sows housed in System I. The indication of better overall health in sows from System I might also be evident in the notably fewer number of medical treatments required for this group during lactation.

System I included separate feeding in lockable stalls enabling individual feed ratios, based on body condition of each sow, and minimized competition over feed. This probably had a positive effect on the general condition of the sows, thus improving the general health of the sows during gestation and lactation. Body condition measurements could have provided valuable insights into how the differing feeding practices and housing environments influenced the overall health and condition of the sows. However, unfortunately such measurements were not available for this study. This may be included in future studies to further enhance our understanding of the interplay between housing conditions, feeding practices, and sow health.

Furthermore, the study acknowledges the potential value of information on the scoring of skin lesions, stereotypic behaviour, and the effects of immediate sow mixing after weaning. While these aspects were not within the scope of this study, recognizing their significance suggests avenues for future investigations to expand our knowledge and enhance the overall assessment of sow welfare and management practices.

## Conclusions

Effective group housing systems for pregnant sows should minimize stress to ensure high productivity and prevent losses from injuries, abortions, and removals. Suggested key features include sufficient space for subordinate animals to avoid aggressions, individual feeding, large floor space allowances when adding new individuals, as well as enrichment materials.

The results indicated that sows provided with larger floor space (totally 156 m^2^ corresponding to 3.9 m^2^ per sow) and housed on deep straw bedding during gestation, exhibited a higher number of liveborn and weaned piglets, and demanded fewer antibiotic treatments than sows housed in pens with less solid floor space (24.5 m^2^ corresponding to 2.5–3.1 m^2^ per sow) and less bedding/ manipulable material.

## Data Availability

No datasets were generated or analysed during the current study.
